# Comparison of methanogen diversity of yak (*Bos grunniens*) and cattle (*Bos taurus*) from the Qinghai-Tibetan plateau, China

**DOI:** 10.1186/1471-2180-12-237

**Published:** 2012-10-19

**Authors:** Xiao Dan Huang, Hui Yin Tan, Ruijun Long, Juan Boo Liang, André-Denis G Wright

**Affiliations:** 1 International Centre for Tibetan Plateau Ecosystem Management, Lanzhou University, Lanzhou 730000, China; 2 Institute of Bioscience, Universiti Putra Malaysia, Serdang 43400 UPM, Malaysia; 3 Institute of Tropical Agriculture, Universiti Putra Malaysia, Serdang 43400 UPM, Malaysia; 4 Department of Animal Science, University of Vermont, 570 Main Street, Burlington, Vermont 05405, USA

## Abstract

**Background:**

Methane emissions by methanogen from livestock ruminants have significantly contributed to the agricultural greenhouse gas effect. It is worthwhile to compare methanogen from “energy-saving” animal (yak) and normal animal (cattle) in order to investigate the link between methanogen structure and low methane production.

**Results:**

Diversity of methanogens from the yak and cattle rumen was investigated by analysis of 16S rRNA gene sequences from rumen digesta samples from four yaks (209 clones) and four cattle (205 clones) from the Qinghai-Tibetan Plateau area (QTP). Overall, a total of 414 clones (i.e. sequences) were examined and assigned to 95 operational taxonomic units (OTUs) using MOTHUR, based upon a 98% species-level identity criterion. Forty-six OTUs were unique to the yak clone library and 34 OTUs were unique to the cattle clone library, while 15 OTUs were found in both libraries. Of the 95 OTUs, 93 putative new species were identified. Sequences belonging to the Thermoplasmatales-affiliated Linage C (TALC) were found to dominate in both libraries, accounting for 80.9% and 62.9% of the sequences from the yak and cattle clone libraries, respectively. Sequences belonging to the Methanobacteriales represented the second largest clade in both libraries. However, *Methanobrevibacter wolinii* (QTPC 110) was only found in the cattle library. The number of clones from the order Methanomicrobiales was greater in cattle than in the yak clone library. Although the Shannon index value indicated similar diversity between the two libraries, the Libshuff analysis indicated that the methanogen community structure of the yak was significantly different than those from cattle.

**Conclusion:**

This study revealed for the first time the molecular diversity of methanogen community in yaks and cattle in Qinghai-Tibetan Plateau area in China. From the analysis, we conclude that yaks have a unique rumen microbial ecosystem that is significantly different from that of cattle, this may also help to explain why yak produce less methane than cattle.

## Background

Yak (*Bos grunniens*) and cattle (*Bos taurus*) separated about 4.4 to 5.3 million years ago [1]. While cattle have a worldwide distribution in most of the low lands, the yak has dominated in high lands especially around the Hindu Kush-Himalayan region and the Qinghai-Tibetan Plateau (QTP), ranging from 3,000 to 5,500 m above sea level. The yak is one of the world’s most remarkable domestic animals, and has been reported as a typical four season grazing ruminant in the QTP [2]. In order to adapt to the harsh environment with severe cold, less oxygen, strong ultra-violet (UV) radiation, and poor forage resources, yaks have evolved special adaptations in physiology, nutrient metabolism and foraging [3-8].

Recently, Shao et al [5] anatomically compared the yak tongue with the cattle tongue, and found that the yak tongue was better adapted to the harsh characteristics of Tibetan pasture. Other recent studies have shown that yaks have an efficient nitrogen metabolism, suggesting an adaptation mechanism to their low-N dietary ingestion under harsh grasslands conditions of the QTP area [8]. Subsequently, using the sulfur-hexafluoride (SF6) tracer technique, Ding et al [9] measured the enteric methane emissions of yak in the QTP area and showed that yaks produce less methane (per unit of live weight) compared to other ruminants, such as cattle.

Greenhouse gases have become a major issue in the world and ruminant livestock are an important source of global enteric methane. Enteric methane gas is produced by microorganisms, called methanogens, in the digestive tract of ruminant livestock during digestion of feed and represents a direct loss of gross energy intake that could more efficiently be used by the animal for increased productivity [10]. Thus, reducing enteric methane production could benefit ruminants energetically, provided that the digestion efficiency and animal production traits are not compromised. The yak has great potential as an “energy-saving” animal as many researchers around the world aim to find “low carbon” livestock.

The identification of inhibitors of methanogenesis is currently being explored. However, the successful use of these agents is dependant upon having a better understanding of the hydrogenotrophic microbial community in the rumen, which must be promoted in the absence of the methanogenic archaea for production benefits to occur. As a potential “low carbon” animal, yaks are adapted to a cold and high altitude environment and are reported to produce less methane than cattle per unit body weight [9]. Thus, the yak, which is well adapted to its environment, may harbor a rumen methanogen population that produces less methane than cattle. Therefore, it is necessary to study the hydrogenotrophic microbial community by comparing the rumen methanogen diversity of yaks and cattle. The phylogenetic analysis of bacterial diversity in yak has been studied previously [11,12], whereas the methanogen diversity in yak has yet to be investigated.

This study aims to generate new knowledge pertaining to the rumen methanogens of the yak and will contribute to the identification of the microbiology that constitutes a low-methane emitting ruminant animal. To our knowledge, this is the first investigation on the diversity of rumen methanogens from the yak.

## Results

### Sequence similarity analysis

In the yak 16S rRNA gene clone library, a total of 227 clones were examined and 18 clones were identified as chimeras and excluded from further analyses. The remaining 209 clones revealed 134 unique sequences (Table 1). Of these, 109 sequences belonged to the Thermoplasmatales-affiliated Lineage C (TALC), with only 85.5% to 89.2% identity to *Methanomassiliicoccus luminyensis*. The remaining 25 sequences were related to archaeal taxa from the orders Methanobacteriales, Methanomicrobiales and Methanosarcinales. Of these 25 sequences, 20 belonged within the order Methanobacteriales and were broken down as follows: 12 sequences were 97.0% to 98.3% related to *Methanobrevibacter millerae,* four sequences had 96.7% to 98.9% identity to *Methanobrevibacter ruminantium,* and four sequences were 96.2% to 97.5% related to *Methanobrevibacter smithii*. Only one sequence was related to methanogens from the order Methanomicrobiales, with 99.8% identity to *Methanomicrobium mobile,* whereas four sequences belonged to the order Methanosarcinales with only 91.7% to 92.9% identity to *Methanimicrococcus blatticola.*

**Table 1:** Similarity values of rumen methanogens from yak and cattle from Qinghai-Tibetan Plateau, China

**Yak**					**Cattle**				
**16S Sequence**	**Clones**^**a**^	**OTU#**	**Nearest Taxon**	**% Seq ID**	**16S Sequence**	**Clones**^**a**^	**OTU#**	**Nearest Taxon**	**% Seq ID**
QTPYAK1	5	74	*Mms. luminyensis*	88.2	QTPC1	2	82	*Mbb. millerae*	98.6
QTPYAK2	1	74	*Mms. luminyensis*	88.1	QTPC2	1	82	*Mbb. millerae*	99.0
QTPYAK3	1	82	*Mbb. millerae*	97.9	QTPC3	3	82	*Mbb. millerae*	98.2
QTPYAK4	1	82	*Mbb. millerae*	98.1	QTPC4	4	49	*Mms. luminyensis*	87.9
QTPYAK5	1	82	*Mbb. millerae*	97.6	QTPC5	1	63	*Mms. luminyensis*	88.4
QTPYAK6	1	82	*Mbb. millerae*	98.3	QTPC6	4	49	*Mms. luminyensis*	87.8
QTPYAK7	2	82	*Mbb. millerae*	98.1	QTPC7	1	33	*Mms. luminyensis*	87.8
QTPYAK8	1	84	*Mbb. millerae*	97.1	QTPC8	1	82	*Mbb. millerae*	99.1
QTPYAK9	1	82	*Mbb. millerae*	98.0	QTPC9	2	82	*Mbb. gottschalkii*	97.6
QTPYAK10	1	82	*Mbb. millerae*	98.2	QTPC10	1	82	*Mbb. millerae*	98.3
QTPYAK11	1	83	*Mbb. millerae*	97.7	QTPC11	1	82	*Mbb. millerae*	98.3
QTPYAK12	1	89	*Mbb. smithii*	96.3	QTPC12	1	82	*Mbb. millerae*	97.7
QTPYAK13	1	50	*Mms. luminyensis*	87.9	QTPC13	1	82	*Mbb. millerae*	98.4
QTPYAK14	2	51	*Mms. luminyensis*	88.8	QTPC14	1	82	*Mbb. millerae*	98.7
QTPYAK15	2	36	*Mms. luminyensis*	87.1	QTPC15	1	82	*Mbb. gottschalkii*	98.4
QTPYAK16	1	52	*Mms. luminyensis*	87.8	QTPC16	1	10	*Mms. luminyensis*	87.1
QTPYAK17	3	49	*Mms. luminyensis*	88.2	QTPC17	3	82	*Mbb. millerae*	98.0
QTPYAK18	1	53	*Mms. luminyensis*	88.0	QTPC18	1	82	*Mbb. millerae*	97.9
QTPYAK19	1	16	*Mms. luminyensis*	87.0	QTPC19	2	82	*Mbb. millerae*	97.9
QTPYAK20	1	68	*Mms. luminyensis*	87.4	QTPC20	1	82	*Mbb. millerae*	98.3
QTPYAK21	1	4	*Mms. luminyensis*	88.0	QTPC21	1	82	*Mbb. millerae*	98.5
QTPYAK22	2	49	*Mms. luminyensis*	88.1	QTPC22	1	82	*Mbb. millerae*	98.4
QTPYAK23	2	49	*Mms. luminyensis*	88.1	QTPC23	2	82	*Mbb. millerae*	97.7
QTPYAK24	2	61	*Mms. luminyensis*	88.4	QTPC24	1	82	*Mbb. millerae*	98.3
QTPYAK25	1	62	*Mms. luminyensis*	88.6	QTPC25	2	82	*Mbb. millerae*	98.1
QTPYAK26	4	49	*Mms. luminyensis*	88.0	QTPC26	2	82	*Mbb. millerae*	97.9
QTPYAK27	1	49	*Mms. luminyensis*	87.8	QTPC27	1	86	*Mbb. smithii*	96.8
QTPYAK28	1	49	*Mms. luminyensis*	88.5	QTPC28	1	49	*Mms. luminyensis*	87.9
QTPYAK29	1	49	*Mms. luminyensis*	87.8	QTPC29	2	28	*Mms. luminyensis*	86.8
QTPYAK30	2	85	*Mbb. smithii*	97.5	QTPC30	6	80	*Mmb. mobile*	99.7
QTPYAK31	2	82	*Mbb. millerae*	98.3	QTPC31	1	80	*Mmb. mobile*	99.7
QTPYAK32	3	88	*Mbb. millerae*	97.0	QTPC32	1	80	*Mmb. mobile*	99.4
QTPYAK33	1	90	*Mbb. millerae*	97.0	QTPC33	3	80	*Mmb. mobile*	99.5
QTPYAK34	1	70	*Mms. luminyensis*	88.5	QTPC34	2	80	*Mmb. mobile*	99.5
QTPYAK35	1	70	*Mms. luminyensis*	88.4	QTPC35	7	80	*Mmb. mobile*	99.8
QTPYAK36	1	70	*Mms. luminyensis*	88.4	QTPC36	4	70	*Mms. luminyensis*	88.0
QTPYAK37	1	70	*Mms. luminyensis*	88.3	QTPC37	3	16	*Mms. luminyensis*	86.6
QTPYAK38	1	77	*Mms. luminyensis*	87.9	QTPC38	5	39	*Mms. luminyensis*	86.6
QTPYAK39	3	70	*Mms. luminyensis*	88.5	QTPC39	9	39	*Mms. luminyensis*	86.5
QTPYAK40	1	70	*Mms. luminyensis*	88.4	QTPC40	2	39	*Mms. luminyensis*	86.7
QTPYAK41	1	70	*Mms. luminyensis*	88.4	QTPC41	1	16	*Mms. luminyensis*	86.5
QTPYAK42	1	70	*Mms. luminyensis*	88.6	QTPC42	3	58	*Mms. luminyensis*	87.8
QTPYAK43	4	74	*Mms. luminyensis*	87.8	QTPC43	2	16	*Mms. luminyensis*	86.7
QTPYAK44	4	74	*Mms. luminyensis*	87.9	QTPC44	3	58	*Mms. luminyensis*	88.3
QTPYAK45	2	74	*Mms. luminyensis*	87.9	QTPC45	4	69	*Mms. luminyensis*	86.6
QTPYAK46	1	71	*Mms. luminyensis*	88.6	QTPC46	1	56	*Mms. luminyensis*	87.9
QTPYAK47	7	81	*Mmc. blatticola*	92.9	QTPC47	1	55	*Mms. luminyensis*	87.9
QTPYAK48	2	81	*Mmc. blatticola*	92.9	QTPC48	1	67	*Mms. luminyensis*	88.6
QTPYAK49	1	81	*Mmc. blatticola*	91.7	QTPC49	1	82	*Mbb. millerae*	98.5
QTPYAK50	2	81	*Mmc. blatticola*	92.6	QTPC50	1	1	*Mms. luminyensis*	87.6
QTPYAK51	2	49	*Mms. luminyensis*	88.7	QTPC51	1	82	*Mbb. millerae*	97.5
QTPYAK52	1	37	*Mms. luminyensis*	88.0	QTPC52	1	82	*Mbb. millerae*	98.3
QTPYAK53	1	57	*Mms. luminyensis*	87.7	QTPC53	1	82	*Mbb. millerae*	98.2
QTPYAK54	2	74	*Mms. luminyensis*	87.9	QTPC55	1	82	*Mbb. millerae*	98.8
QTPYAK55	1	76	*Mms. luminyensis*	87.1	QTPC56	1	25	*Mms. luminyensis*	86.7
QTPYAK56	2	72	*Mms. luminyensis*	87.5	QTPC57	1	41	*Mms. luminyensis*	86.4
QTPYAK57	1	72	*Mms. luminyensis*	87.6	QTPC58	2	94	*Mbb. millerae*	96.0
QTPYAK58	2	72	*Mms. luminyensis*	87.9	QTPC59	2	55	*Mms. luminyensis*	87.8
QTPYAK59	1	75	*Mms. luminyensis*	87.3	QTPC60	4	55	*Mms. luminyensis*	87.7
QTPYAK60	1	70	*Mms. luminyensis*	88.1	QTPC61	2	55	*Mms. luminyensis*	87.8
QTPYAK61	1	39	*Mms. luminyensis*	86.3	QTPC62	1	73	*Mms. luminyensis*	87.6
QTPYAK62	2	39	*Mms. luminyensis*	86.2	QTPC63	1	41	*Mms. luminyensis*	86.5
QTPYAK63	2	39	*Mms. luminyensis*	86.5	QTPC64	1	91	*Mbb. millerae*	96.1
QTPYAK64	4	46	*Mms. luminyensis*	86.7	QTPC65	1	73	*Mms. luminyensis*	87.5
QTPYAK65	1	49	*Mms. luminyensis*	88.4	QTPC66	1	40	*Mms. luminyensis*	87.4
QTPYAK67	2	80	*Mmb. mobile*	99.8	QTPC68	1	7	*Mms. luminyensis*	87.4
QTPYAK68	1	64	*Mms. luminyensis*	87.5	QTPC69	1	82	*Mbb. millerae*	98.6
QTPYAK69	2	93	*Mbb. ruminantium*	96.7	QTPC70	1	94	*Mbb. arboriphilus*	95.5
QTPYAK70	1	87	*Mbb. ruminantium*	96.8	QTPC71	1	59	*Mms. luminyensis*	88.9
QTPYAK71	1	87	*Mbb. smithii*	96.5	QTPC72	1	59	*Mms. luminyensis*	89.2
QTPYAK72	1	32	*Mms. luminyensis*	86.8	QTPC73	3	1	*Mms. luminyensis*	87.8
QTPYAK73	1	92	*Mbb. ruminantium*	98.1	QTPC74	10	16	*Mms. luminyensis*	86.6
QTPYAK74	1	92	*Mbb. ruminantium*	98.9	QTPC75	1	16	*Mms. luminyensis*	86.5
QTPYAK75	1	35	*Mms. luminyensis*	87.2	QTPC76	2	16	*Mms. luminyensis*	86.6
QTPYAK76	1	49	*Mms. luminyensis*	88.4	QTPC77	6	16	*Mms. luminyensis*	86.6
QTPYAK77	1	42	*Mms. luminyensis*	88.3	QTPC78	1	16	*Mms. luminyensis*	86.6
QTPYAK78	1	42	*Mms. luminyensis*	87.5	QTPC79	1	24	*Mms. luminyensis*	87.0
QTPYAK79	1	16	*Mms. luminyensis*	86.6	QTPC80	1	16	*Mms. luminyensis*	86.2
QTPYAK80	1	16	*Mms. luminyensis*	86.7	QTPC81	1	16	*Mms. luminyensis*	86.7
QTPYAK81	10	16	*Mms. luminyensis*	86.6	QTPC82	1	20	*Mms. luminyensis*	83.8
QTPYAK82	1	16	*Mms. luminyensis*	86.5	QTPC83	1	9	*Mms. luminyensis*	87.6
QTPYAK83	1	16	*Mms. luminyensis*	86.4	QTPC84	1	24	*Mms. luminyensis*	86.4
QTPYAK84	3	16	*Mms. luminyensis*	86.4	QTPC85	1	26	*Mms. luminyensis*	86.4
QTPYAK85	1	16	*Mms. luminyensis*	86.4	QTPC86	2	48	*Mms. luminyensis*	87.3
QTPYAK86	1	16	*Mms. luminyensis*	86.7	QTPC87	1	21	*Mms. luminyensis*	86.8
QTPYAK87	1	16	*Mms. luminyensis*	86.7	QTPC88	1	23	*Mms. luminyensis*	86.3
QTPYAK88	1	16	*Mms. luminyensis*	87.0	QTPC89	1	22	*Mms. luminyensis*	86.4
QTPYAK89	1	16	*Mms. luminyensis*	86.6	QTPC90	1	39	*Mms. luminyensis*	87.3
QTPYAK90	1	16	*Mms. luminyensis*	86.7	QTPC91	1	42	*Mms. luminyensis*	87.9
QTPYAK91	2	16	*Mms. luminyensis*	86.5	QTPC92	1	2	*Mms. luminyensis*	86.9
QTPYAK92	1	16	*Mms. luminyensis*	87.4	QTPC93	1	2	*Mms. luminyensis*	88.0
QTPYAK93	1	16	*Mms. luminyensis*	87.2	QTPC94	1	1	*Mms. luminyensis*	87.7
QTPYAK94	6	16	*Mms. luminyensis*	86.5	QTPC95	6	81	*Mmc. blatticola*	92.8
QTPYAK95	2	16	*Mms. luminyensis*	86.3	QTPC96	6	81	*Mmc. blatticola*	92.5
QTPYAK96	2	16	*Mms. luminyensis*	87.2	QTPC97	2	39	*Mms. luminyensis*	87.1
QTPYAK97	1	16	*Mms. luminyensis*	86.3	QTPC98	1	39	*Mms. luminyensis*	87.2
QTPYAK98	1	15	*Mms. luminyensis*	87.2	QTPC99	1	47	*Mms. luminyensis*	86.4
QTPYAK99	1	27	*Mms. luminyensis*	87.1	QTPC100	1	59	*Mms. luminyensis*	88.5
QTPYAK100	1	27	*Mms. luminyensis*	87.4	QTPC101	1	79	*Mms. luminyensis*	87.1
QTPYAK101	1	14	*Mms. luminyensis*	87.0	QTPC102	1	5	*Mms. luminyensis*	88.4
QTPYAK102	1	24	*Mms. luminyensis*	86.7	QTPC103	1	6	*Mms. luminyensis*	87.6
QTPYAK103	1	12	*Mms. luminyensis*	87.3	QTPC104	1	66	*Mms. luminyensis*	88.5
QTPYAK104	1	19	*Mms. luminyensis*	85.5	QTPC105	1	29	*Mms. luminyensis*	86.4
QTPYAK105	1	13	*Mms. luminyensis*	87.5	QTPC106	1	45	*Mms. luminyensis*	87.4
QTPYAK106	1	17	*Mms. luminyensis*	85.9	QTPC107	1	54	*Mms. luminyensis*	87.7
QTPYAK107	1	17	*Mms. luminyensis*	86.4	QTPC108	1	48	*Mms. luminyensis*	86.7
QTPYAK108	1	11	*Mms. luminyensis*	86.8	QTPC109	1	30	*Mms. luminyensis*	86.5
QTPYAK109	3	16	*Mms. luminyensis*	86.5	QTPC110	1	95	*Mbb. wolinii*	95.7
QTPYAK110	1	18	*Mms. luminyensis*	86.2	QTPC111	1	39	*Mms. luminyensis*	86.3
QTPYAK111	1	16	*Mms. luminyensis*	86.8	QTPC112	1	92	*Mbb. ruminantium*	99.0
QTPYAK112	2	16	*Mms. luminyensis*	85.9	QTPC113	1	43	*Mms. luminyensis*	88.4
QTPYAK113	1	18	*Mms. luminyensis*	86.3	QTPC114	1	42	*Mms. luminyensis*	87.7
QTPYAK114	2	16	*Mms. luminyensis*	86.2					
QTPYAK115	1	16	*Mms. luminyensis*	86.3					
QTPYAK116	1	34	*Mms. luminyensis*	87.2					
QTPYAK117	2	34	*Mms. luminyensis*	87.7					
QTPYAK118	1	8	*Mms. luminyensis*	88.1					
QTPYAK119	2	34	*Mms. luminyensis*	87.9					
QTPYAK120	1	41	*Mms. luminyensis*	86.3					
QTPYAK121	1	89	*Mbb. smithii*	96.2					
QTPYAK122	1	44	*Mms. luminyensis*	87.9					
QTPYAK123	1	58	*Mms. luminyensis*	87.9					
QTPYAK124	1	78	*Mms. luminyensis*	88.1					
QTPYAK125	1	59	*Mms. luminyensis*	89.1					
QTPYAK126	1	59	*Mms. luminyensis*	89.2					
QTPYAK127	1	74	*Mms. luminyensis*	88.1					
QTPYAK128	1	2	*Mms. luminyensis*	87.7					
QTPYAK129	2	38	*Mms. luminyensis*	88.2					
QTPYAK130	1	65	*Mms. luminyensis*	88.7					
QTPYAK132	1	58	*Mms. luminyensis*	88.9					
QTPYAK133	1	60	*Mms. luminyensis*	88.7					
QTPYAK134	1	2	*Mms. luminyensis*	87.3					
QTPYAK135	1	21	*Mms. luminyensis*	87.1					

*Mbb.= Methanobrevibacter; Mms=Methanomassiliicoccus; Mmb=Methanomicrobium; Mmc=Methanimicrococcus*.

*16S Sequences were obtained from MOTHUR program as unique sequences, while OTUs were generated by the MOTHUR program at 98% species level identity.

In the cattle 16S rRNA gene library, a total of 216 clones was examined, of which 11 clones were identified as chimeras and excluded from the analysis. The remaining 205 sequences revealed 113 unique sequences (Table 1). A total of 72 sequences (129 clones) were only 83.8% to 89.2% related to *Methanomassiliicoccus luminyensis*, whereas 33 sequences (44 clones) were 95.5% to 99.1% related to methanogens belonging to the order Methanobacteriales and six sequences (20 clones) were 99.4 to 99.8% related to those belonging to the order Methanomicrobiales. The remaining two sequences (12 clones) were 92.5% and 92.8% related to *Methanimicrococcus blatticola* within the order Methanosarcinales. Within the Methanobacteriales, 27 of the 33 sequences were 96.0% to 99.1% identical to *Methanobrevibacter millerae*, two sequences (QTPC 9 and QTPC 15) were 97.6 to 98.4% related to *Methanobrevibacter gottschalkii*; one sequence (QTPC 70) was only 95.5% related to *Methanobrevibacter arboriphilus*; and three sequences (QTPC 112, QTPC 27 and QTPC 110) were 99%. 96.8% and 95.7% related to *Methanobrevibacter ruminantium*, *Methanobrevibacter smithii* and *Methanobrevibacter wolinii*, respectively.

Using a species-level identity criterion of 98% [13], 93 of the 95 OTUs had less than 98% identity to any valid recognized taxa, and may represent potential new methanogen species and strains.

### Statistical analysis of libraries

The yak library had a Shannon index of 3.33±0.18 while the cattle library had a Shannon index of 3.02±0.19. Libshuff analysis showed that the differences between the yak and cattle libraries at 98% identity were significant (*P<* 0.0001).

### Phylogenetic placement of sequences

**Figure 1:**
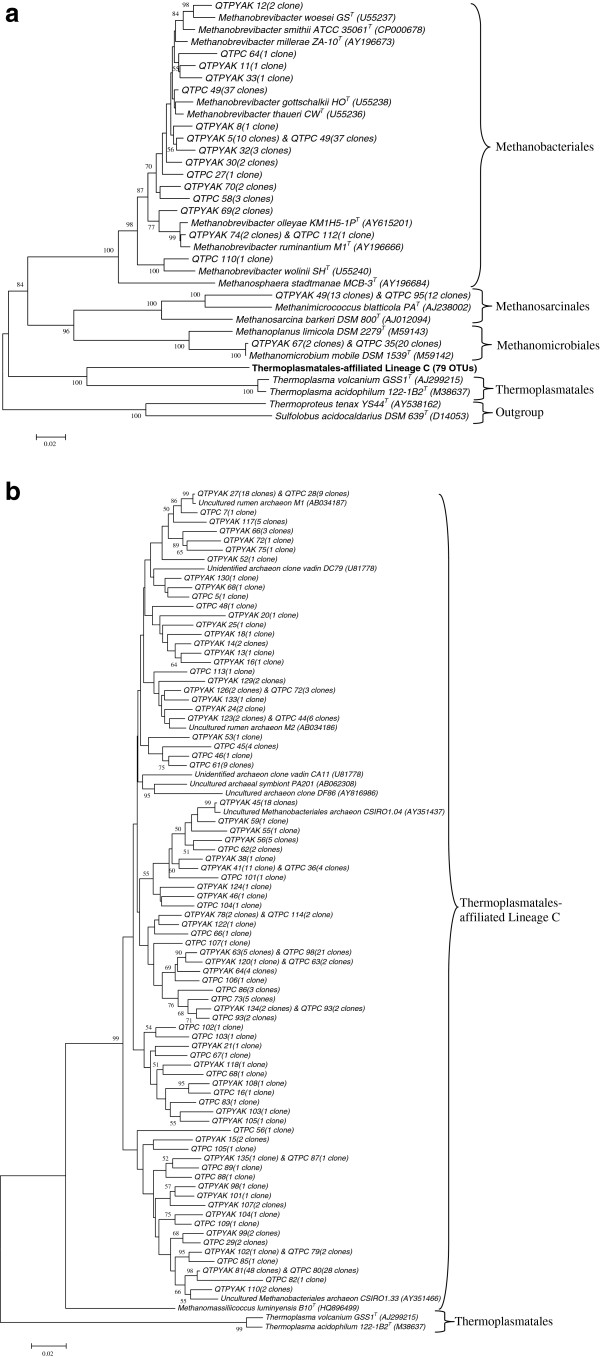
**Phylogenetic analysis of methanogen partial 16S rRNA sequences from yak and cattle clone library inferred using MEGA (ver. 5).** Of the 414 clones examined, 209 clones from yak and 205 clones from cattle were assigned to 95 OTUs by MOTHUR using a 98% species level identity. These 95 OTUs are shown by representative sequences on the tree. In which, 16 OTUs from non-TALC group are presented in Figure 1a, and 79 OTUs from TALC group are presented in Figure 1b. GenBank accession number are indicated in parentheses and bootstrap values (>50%) from 1000 replications are indicated on the tree.The scale bar corresponds to 2 changes per 100 positions.

Distance-matrix phylogenetic trees are provided showing the phylogenetic placement of the methanogen sequences from the yak and cattle (Figure 1) clone libraries. Methanogen sequences from yak and cattle grouped with methanogens from the uncharacterized TALC group (Figure 1b), as well as the orders Methanobacteriales, Methanomicrobiales, Methanosarcinales (Figure 1a).

In total, 414 clones were analyzed, revealing 247 unique sequences (134 sequences from yak and 113 sequences from cattle), which were assigned to 95 OTUs (79 TALC and 16 non-TALC). Examination of these 95 OTUs revealed that, 46 OTUs were unique to the yak clone library and 34 OTUs were unique to the cattle clone library (Figure 1a and 1b), while 15 OTUs (15.8%) were found in both libraries as shared OTUs.

## Discussion

The Yak is a key species in the Qinghai Tibetan Plateau. It provides herders with milk, meat, fiber, fuel and draught power, but also plays a key role in the management of the alpine rangeland ecosystem [14]. This ecological niche is unique and no other animal species can substitute the yak at such harsh environments (i.e. high altitude with lower oxygen levels and freezing temperatures in the winter). Research on the yak production system is therefore highly strategic and in recent years, adaptations of physiology, nitrogen and energy metabolism, histological variations, and foraging behavior to the harsh forage environment have been revealed [3-8]. However, research focusing on the rumen microbiota of the yak, has been limited until now. Based upon the Libshuff analysis, the current study has shown that the community structure of the methanogens resident in the yak is significantly different (p<0.0001) from that of cattle, with only 15 of the 95 OTUs shared between the two libraries.

The rumen is a unique environment which inhabits billions of microorganisms, including bacteria, methanogenic archaea, protozoa and fungi. Common species of methanogens isolated from rumen belong to the genera, *Methanobrevibacter*, *Methanomicrobium*, *Methanobacterium* and *Methanosarcina*[15,16]. In the present study, the majority of methanogen sequences were very distantly related to *Methanomassiliicoccus luminyensis* (Table 1) and were found to belong to the Thermoplasmatales-affiliated Lineage C, a group of uncultivated and uncharacterized rumen archaea that is a distantly related sister group to the order Thermoplasmatales (Figures 1). Tajima et al [17] also reported the methanogen diversity of the bovine rumen exhibited high degrees of similarity to uncultured archaea which were distantly related to the order Thermoplasmatales. Wright et al [18] also reported that 18 of 26 unique sequences from Australia sheep had 72 to 75% identity to Thermoplasmatales and were considered as predominant sequences in the rumen. In present study, within the TALC clade, few unique OTUs from yak and cattle libraries were highly related to the clones M1and M2 from Holstein cattle in Japan [17], clones CSIRO 1.04 and CSIRO 1.33 from sheep in Western Australia [18], and clones vadin CA11 and vadin DC79 from a wine anaerobic digester in France [19].

The distribution of 16S rRNA gene sequences within the orders of Methanobacteriales and Methanomicrobiales also varied between yak and cattle clone libraries. From the results, it was apparent that a greater percentage of the methanogen population from the orders of Methanobacteriales (21.5% vs 12.4%) and Methanomicrobiales (9.8% vs 0.96%) were found in the rumen of cattle as compared to the yak.

Zhou et al [20] studied the methanogen diversity in cattle with different feed efficiencies and reported that differences at the strain and genotype levels of metagenomic ecology were found to be associated with feed efficiency in the host regardless of the population of methanogens. It was also suggested that the microbial ecology at the species, strain and genus levels in the rumen may play important roles in contributing to the difference in the methane gas production.

A recent investigation found that condensed tannins could exhibit a reduction in methane production in an *in vitro* gas production test [21]. Further investigation into the diversity of 16S rRNA gene library of rumen methanogen in the condensed tannin treatment library revealed 21.9% higher diversity of sequences related to the TALC methanogens and a lower diversity of those associated with orders Methanobacteriales (15.1%) and Methanomicrobiales (6.8%) [22]. This shows a possible association between reduction in methane production and diversity of rumen methanogen. In the current study, yak has present higher methanogen diversity and significant different methanogen community structures compared with cattle (Figure 1). While there are many factors which may explain these differences in methanogen diversity, it is possible that these differences between the methanogen diversity in yak and cattle could be related to the significant difference in enteric methane production by both these ruminant species.

Long [23] reported a significantly high level of propionic acid, which leads to efficient energy utilization and this further suggested a low methane production in yak. Yak has also been found to exhibit lower methane output [9]. In the present study, yak had higher levels of acetate, proprionate, isobutyric, isovaleric and total volatile fatty acids than cattle, but cattle had higher acetate to proprionate (A/P) ratios (Table 2). This may also suggest different methanogenesis pathways. Therefore, the diversity and community structure of methanogens in yak, which is the lower methane producing ruminant species in current study, correlates with data reported by Tan et al [22].

**Table 2:** The concentrations of volatile fatty acids from yak and cattle rumen samples

**Volatile fatty acids**	**Yak (mmol/L)**	**Cattle (mmol/L)**	**Standard error**	**Significance**
Acetate	58.56	42.57	3.18	p < 0.004
Propionate	12.13	7.35	0.93	p < 0.001
Isobutyric	0.88	0.60	0.06	p < 0.016
Butyrate	9.03	7.25	0.49	p < 0.09
Isovaleric	1.02	0.51	0.12	p < 0.027
Valeric	0.07	0.13	0.06	p < 0.728
Total volatile fatty acids	81.69	58.41	4.61	p < 0.001
A/P (Acetate to Propionate)	4.83	5.80	0.19	p < 0.004

* Concentrations of volatile fatty acids was analysed by gas chromatograph equipped with a DB-FFAP column (30 m × 0.25 μm × 0.25 μm; Agilent Technologies).

Wright et al [24] revealed 65 sequences of methanogens by phylogenetic analysis from the Australian sheep rumen, and 62 of them belonged to the genus *Methanobrevibacter*. They were grouped with *Methanobrevibacter* NT7, *Methanobrevibacter* SM9, *Methanobrevibacter* M6, *Methanobrevibacter ruminantium*, *Methanobrevibacter acididurans* and *Methanobrevibacter thaueri*. From the present study (Figures 1a and 1b), clones related to *Methanobrevibacter olleyae*, *Methanobrevibacter ruminantium*, *Methanobrevibacter woesei*, *Methanobrevibacter smithii*, *Methanobrevibacter millerae*, *Methanobrevibacter gottschalkii*, and *Methanobrevibacter thaueri* were reported in the yak. However, in contrast with the yak library, *Methanobrevibacter wolinii* was only found in the cattle library. Clones related to *Methanimicrococcus blatticola* and *Methanomicrobium mobile* were found in both libraries.

Bacteria and methanogens has constantly interacted with each other in the rumen microbial communities [25], Sustainable growth of bacteria and methanogen in syntrophic communities depend on transfer of hydrogen and formate and reverse electron transfer [26]. In the present study, methanogens from the TALC cluster were the dominant sequences in the yak and cattle rumen in the QTP area. However, the metabolic mechanism of this methanogen group is not yet clear; the investigation of fermentive bacteria species in yak and cattle could help understanding these syntrophic microbial communities.

## Conclusions

The current study revealed for the first time the molecular diversity of methanogen community in yaks and cattle in Qinghai-Tibetan Plateau area in China. The differences in methanogen diversity found in the present study, may help to explain, to some extent, the differences associated with the low methane production contributed to the adaptation of the yak to the harsh forage environment in the Qinghai-Tibetan plateau. Yaks have co-evolved with a unique rumen microbial ecosystem that is significantly different from that of cattle, even when feed similar diets. Understanding these particularities will yield development of technology for reducing methane emission intensity by optimizing dietary conditions to exploit the full potential of the yak ruminal ecosystem and function. However, native grazing might be a limited factor for this experiment, since feed intake could significantly influence the rumen microbiota. This study also contributes to the understanding of the specific features of the rumen microbial ecosystem of yaks which have adapted to high altitude ecosystems which may help to explain the differential rates of methanogenesis compared to cattle.

## Methods

### Animals and diet

Samples of individual rumen contents were obtained from four domestic cattle (BW: 160 ± 5kg, Age: 4 ± 0.4 years) and four domesticated yaks (body weight: 180 ± 5 kg, Age: 4 ± 0.6 years) in the Qinghai Tibetan Plateau (QTP) in China. The animals were maintained outdoors, grazing a Kobresia pasture. Approximately 100 ml of rumen contents were collected using a 1.5 cm diameter stomach tube attached to an electric pump. The animal sampling procedure strictly followed the rules and regulations of experimental field management protocols (file No: 2010–1 and 2010–2) which were approved by the Lanzhou University. Rumen contents were filtered through four layers of sterilized gauze and the rumen digesta was immediately transferred into sterile bottles and stored in liquid nitrogen until needed for DNA extraction and volatile fatty acids concentration analysis (Table 2).

### DNA extraction and PCR

Genomic DNA was extracted from 300 μl aliquots of the eight (4 yak and 4 cattle) thawed rumen samples using the QIAamp® DNA Stool kit (QIAGEN, Germany). The DNA extraction procedure was carried out in triplicate.

The methanogen-specific primers, Met86F (5^′^- GCT CAG TAA CAC GTG G-3^′^) [27] and Met1340R (5^′^- CGG TGT GTG CAA GGA G-3^′^) [27] were used to PCR amplify the 16S rRNA gene using the following thermal cycling conditions: initial denaturation of 5 min at 94°C, 40 cycles of denaturation at 94°C for 30 s, annealing at 58°C for 1 min, extension at 72°C for 90 s, and a final extension at 72°C for 10 min. Each PCR mixture contained 1 μl (20ug) of genomic DNA, 200 nM of each primer, 10 μM of dNTP (i-DNA Biotechnology Pte Ltd, Singapore), 1x VioTaq® reaction buffer, 0.5 U of VioTaq® Taq DNA polymerase (Viogene, Taiwan) and deionized water, in a final volume of 20 μl. PCR product of about 1.3 kb was isolated from the agarose gel and purified using MEGAquick-spin™ PCR and an agarose gel DNA extraction Kit (iNtRON Biotechnology, Seongnam, South Korea).

### Cloning, sequencing, and analyses

Using chemical transformation, purified PCR products were cloned into the pCR 2.1® TOPO vector using the PCR 2.1® TOPO TA Cloning Kit (Invitrogen Ltd, USA). Recombinant colonies were picked and plasmid DNA was extracted using DNA-spin™ Plasmid DNA Extraction Kit (iNtRON Biotechnology, Korea). Sequencing was performed with an automated sequencer ABI 3730 xl using Big Dye Chemistry.

All sequences were aligned with ClustalW [28] in BioEdit software, and the Basic Local Alignment Search Tool (BLAST) [29] was used to determine the identity to the nearest recognized species available in the GenBank database. A species-level cutoff of 98% [13] was used to assign sequences to OTUs and chimeras were identified using the Mallard program [30].

MOTHUR ver. 1.23.1 [31] was used to assign sequences to OTUs, and within MOTHUR, the Shannon index [32] and Libshuff analysis were used to assess the methanogen diversity and community structure of each library, respectively.

### Phylogenetic analysis

A total of 27 archaeon sequences from GenBank were used as reference sequences, and two members of the *Crenarchaeota*, *Sulfolobus acidocaldarius* (D14053) and *Thermoproteus tenax* (AY538162), were the outgroup. All 16S rRNA gene clone sequences and the reference sequences were globally aligned using CLUSTAL W [33]. Phylogenetic analysis was performed by using MEGA ver 5.0 [34] using the neighbor-joining algorithm [35], with 1,000 bootstrap resamplings of the dataset [36]. Evolutionary distances between pairs of nucleotide sequences were calculated using Kimura two-parameter model [37].

### Nucleotide accession numbers

Nucleotide sequences were designed with the prefix QTPYAK (Qinghai-Tibetan Plateau Yak) to represent 16S rRNA gene sequences from the yak clone library, and QTPC (Qinghai-Tibetan Plateau Cattle) for those from the cattle clone library. Prefixes were followed by the identification number of the unique sequence. All nucleotide sequences reported in this paper have been deposited in the GenBank database under the accession numbers JF807063 to JF807176 (i.e. cattle clones), excluding JF807116 (identical to JF807120); and JF807177 to JF807311 (i.e. Yak clones), excluding JF807307 (identical to JF807305).

## Competing interests

The authors declare that they have no competing interests.

## Authors’ contributions

XDH sampled rumen contents from animals, performed DNA extractions, PCR amplification of methanogen 16S rRNA genes, clone library construction, data analysis, and drafted the manuscript. HYT contributed to all of the lab works and drafted the manuscript. RL conceived the study, sampled rumen contents from animals and drafted the manuscript. JBL contributed to the design of the study and drafted the manuscript; ADW performed data analysis, and drafted the manuscript. All authors read and approved the final manuscript.
